# How the Physical Environment Shapes the Microbiota

**DOI:** 10.1128/mSystems.00675-21

**Published:** 2021-08-24

**Authors:** Carolina Tropini

**Affiliations:** a School of Biomedical Engineering, University of British Columbia, Vancouver, Canada; b Department of Microbiology and Immunology, University of British Columbia, Vancouver, Canada; c Humans and the Microbiome Program, Canadian Institute for Advanced Research (CIFAR), Toronto, Canada

**Keywords:** gut biogeography, microbiota, physical perturbations

## Abstract

Living systems, from micro- to macro-scales, are strongly impacted by physical factors such as temperature, pH, and the concentration of compounds in their surrounding environment. In the macro-world, it is obvious that small changes in these parameters can have profound, and even devastating, impacts on an ecosystem. For example, in the case of global warming, a change in climate, and specifically a few degrees in temperature, has taken one million species of animals to the brink of extinction. Scale things down 6 orders of magnitude, our gut microbiota also experiences similar changes in temperature due to disease. In this highly competitive environment, physical perturbations inflict long-term consequences on the microbiota ecosystem and, in turn, on the host organism. My laboratory is exploring the feedback between the gut’s physical environment, the microbiota, and disease. Our research highlights the importance of measuring physical parameters for the prediction of microbial dynamics and microbiota therapies.

## COMMENTARY

When I think of the physical environment and its effect on living systems, I cannot avoid reflecting on global warming, and how many animal and plant species we are at risk of losing (the estimate is a staggering one-third [1]). As a naive biophysicist entering the microbiology field during my Ph.D., I realized that living systems at all scales, from plants and animals down to microbes, are impacted by the physical environment. For example, we normally think of a fever as a short, transient illness, but it affects the trillions of microorganisms in our gut over tens of their generations, similar generational timescales as those on which global warming impacts mammals. In the macro-scale world, global warming affects climate and weather; could this be akin to a fever’s impact on gut motility and host responses? Following the analogy, could a fever lead to the loss of microbial species in our gut?

Our gut microbiota functions as a personalized pharmacy in our gut—anything our microbes excrete has the potential to make it through our blood system and impact all organs, just like the compounds in a pill taken orally. Can major changes in the gut environment modify the availability of these compounds, and hence human health? My laboratory is addressing these questions by investigating how physical factors due to natural physiology, disease, and industrialization affect the gut microbiota and host health.

## PHYSICAL FACTORS SHAPE OUR GUT-ASSOCIATED MICROBIOTA

Physical factors are important to microbes living in host-associated environments but are often overlooked. Here I am referring to physical factors as features of an ecosystem that lead to physical forces. For example, a sugar such as glucose has a biochemical impact on a microbial ecosystem, but it’s also a solute that impacts the osmotic pressure of the habitat. Similarly, while short chain fatty acids are crucial microbial metabolites, they are also key molecules affecting gut pH balance and hydrogen potential.

We usually focus on the biochemical interactions between microbes and their host. These are of course critical—understanding metabolic function is essential to achieving predictability in these complex communities. However, just like at the macro-scale, the physical environment sets fundamental ground rules that can supersede biochemical interactions.

**FIG 1:**
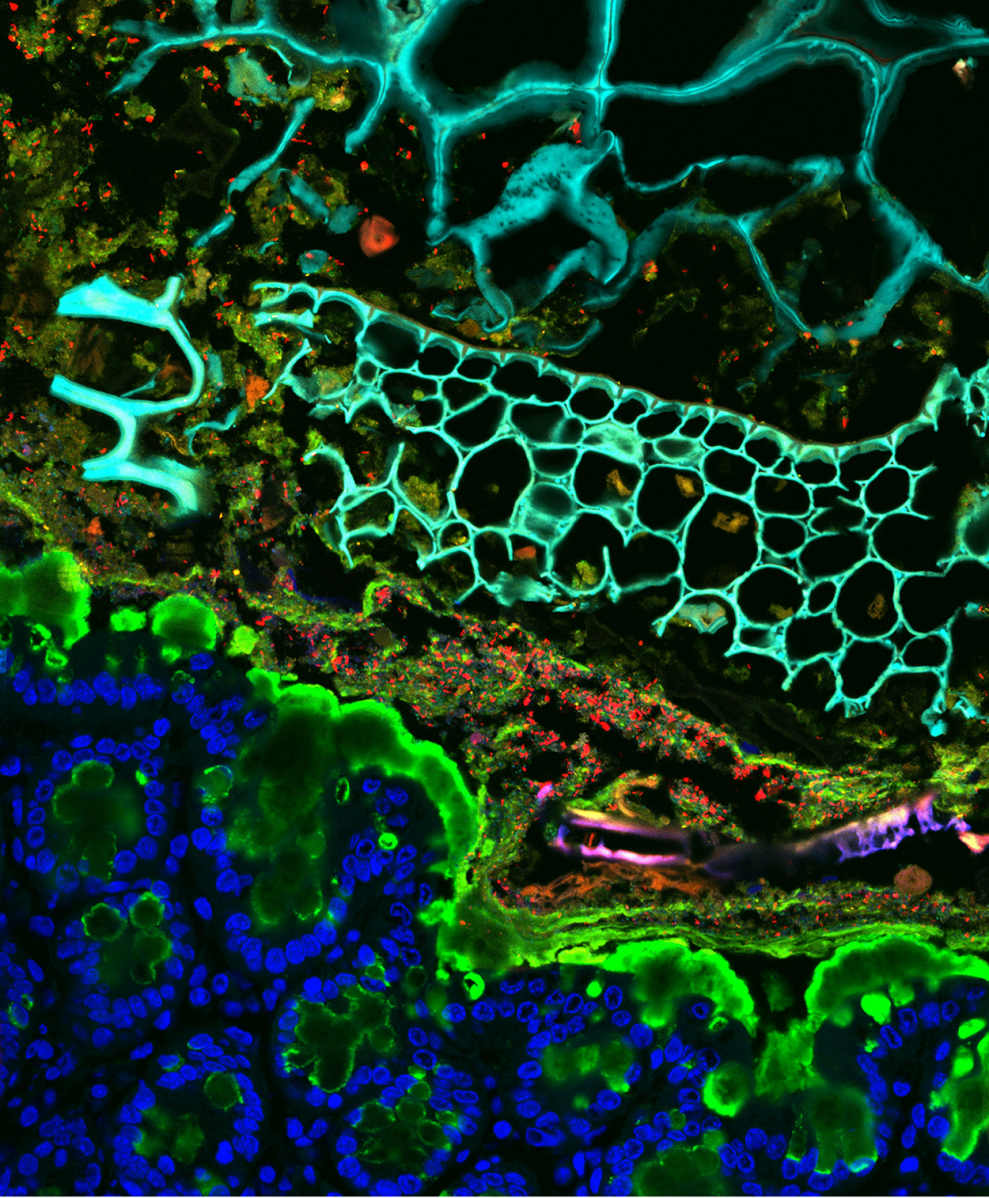
*In situ* imaging of the gastrointestinal tract highlights a complex environment. Intestinal sections were stained with 4′,6-diamidino-2-phenylindole (DAPI) (false colored blue in tissue and cyan in lumen) and labeled with fluorescence *in situ* hybridization (FISH) probes (red, *Bacteroidaceae*; green, *Enterococcus*) and UEA-1 (green) to label mucus.

The intestinal gut environment is determined by multiple physical parameters, including osmolality, pH, flow, and mucus stiffness, among many others; these parameters vary along the length of the gut and are tightly regulated by gut epithelial absorption and secretion. The importance of gut biogeography and physical factors in the establishment of bacterial communities is well documented (2–4), but it has been minimally explored as a driver of community composition. The physical environment is particularly important in the limiting cases—e.g., bacteria that are unable to grow at low pH won’t be able to take advantage of the high nutrient concentration in the stomach. Physical niches in the intestines are carefully modulated by a myriad of host secretions such as bile acids, gastric fluid, and bicarbonate (5–7). The microbiota itself is also able to feedback on the physical environment via fermentation products and excretion of compounds such as short-chain fatty acids. Thanks to all of these factors and feedbacks, the pH of a healthy gut naturally varies along the digestive system by as much as 4 logs (5). Therefore, even without considering the changing biochemical and immunological niches, microbes are in steep competition to grow in selected locations in their journey through the gut. By the time bacteria are excreted as stool, this rich history of exploration through multiple complex environments is all but lost. Only via *in situ* measurements can we start to appreciate the complexity of these ecosystems, comprising multiple habitats within a single gastrointestinal tract. In our lab we leverage physical measurements of the gut environment paired with imaging and spatial measurements to explore this complexity at the single-cell level (Fig. 1). If the gut environment is heterogeneous in health, what happens during disease?

## DISEASE RESHAPES THE GUT ENVIRONMENT

As one might expect, while the heterogeneity of the gut environment is tightly regulated in health, this control can be largely lost during host illness. Disease can cause local and systemic changes to the gut physical environment such as changes in pH in inflammatory bowel disease (IBD) (5), changes in osmolality and flow due to malabsorption and diarrhea (8), or changes in temperature due to inflammation (9, 10). These changes in the physical environment naturally impact the resident microbiota and apply strong selective pressures. Beyond constraining the ability of organisms to grow within an environment, physical stressors also greatly impact cellular physiology even in regimes where growth can occur. For example, hyperosmotic stress, as caused by malabsorption in the gut, leads to the outflow of water into the gut lumen (from epithelial and bacterial cells alike) to compensate for the increased external molecule concentration. Water excretion mechanically impacts the bacterial envelope due to reduced turgor pressure inside the cell. Furthermore, reduced water in the cytoplasm changes molecular interactions because of increased crowding. Which microbes best survive these changes? Unfortunately for the host, it’s the pathogens, which tend to have a wider arsenal of stress response pathways than commensal bacteria. Infectious agents can even reinforce the disrupted habitat, further hampering recovery of the microbiota and gut health (which we recently reviewed in the context of gut biogeography [11]). Diseased states can become stable and lead to chronic conditions where homeostasis is not retained by the host. Unsurprisingly, loss of the natural gut equilibrium feeds back on the microbiota. Importantly, our lab is finding that the impact of chronic diseases cannot be unraveled without comprehending how the changed physical environment sets up changed habitats affecting drug delivery and the ability of microbes to colonize, grow, and evolve.

## EVOLUTION AND ERADICATION IN THE MICROBIOTA

When physical stresses are prolonged, microbes may need to modify complex cellular components, such as the cell wall in the case of osmotic stress, to adapt. These adaptations are harder to achieve than modifying individual proteins to develop resistance (e.g., an uptake channel in the case of response to an antibiotic). This means that the physical environment can strongly limit the capacity of an organism to persist within a competitive habitat because of the complexity of the adaptations that are required.

Importantly, even short perturbation timescales from the perspective of the host affect many generation cycles for the microbiota. Supporting this idea, during my postdoc, as I was analyzing my first microbiome data set, I found that increasing gut osmolality by inducing malabsorption over a period of days led a single family comprising almost 50% of the bacteria to disappear (12). Had I made a mistake? How could such a short perturbation lead to such a devastating effect? After repeating this experiment in multiple different communities, I realized that we were witnessing the wiping out of an entire family of bacteria from an ecosystem due to a change in the physical environment. I remember trying to explain to my family how big of an effect this is. Think of taking away all felines or primates from a habitat—this is what eradicating a family of organisms is like at the macro-scale.

We later demonstrated that members of this family, called *Muribaculaceae* (or S24-7), are highly sensitive to increased osmolality *in vitro*, leading them to disappear during osmotic laxative treatment *in vivo* and not return postrecovery (12). Interestingly, upon external reintroduction, we were able to reestablish this family in the gut, but only provided that the osmolality levels were normalized (12), highlighting the importance of the physical environment for microbiota colonization as well as maintenance. As we started following up on this work, we wondered, what happens if lost microbes are not externally reintroduced?

## ARE WE CHANGING OUR MICROBIOTA FOR THE WORSE?

A major driver for changes in the physical environment at the macro-scale is industrialization. Over the past 150 years, industrialization has impacted living systems at all scales, with lasting effects. We have heavily modified our gut habitat because we have changed the way we eat, fight disease, and interact with our environment. Parallel to macro-scale physical environment changes such as global warming and acidification, modern drugs such as antacids and laxatives can significantly disrupt the micro-scale physical environment within our bodies. Nearly all efforts to study the impact of the gut microbiota in health and disease have focused on the effect of microbes present in the microbiota; however, recent strong evidence also points to the critical role for foundational microbial members missing due to lifestyle changes and industrialization (13, 14). Reduced gut microbial diversity has been linked to numerous “modern” diseases such as inflammatory bowel disease, obesity, allergies, and autoimmune disorders (15). My lab is deeply interested in understanding how changes to the physical environment due to modern diseases lead to the disappearance of beneficial microbes, and how to counteract those changes.

As an example, the *Muribaculaceae* family, which we found to be so negatively impacted by osmotic laxatives, was named as the most prevalent proximal gut bacterium in homeothermic animals (16); however, it was found in only 10% of individuals in industrialized human populations (17). Interestingly, *Muribaculaceae* are highly prevalent in traditional uncontacted Amerindians (18) as well as the Hadza in Tanzania (19), indicating that they may be vanishing in industrialized countries due to changes in lifestyle. There is accumulating evidence that *Muribaculaceae* presence is anticorrelated from certain industrialized world diseases such as diabetes (20, 21), and its disappearance correlates with the increasing prevalence and incidence of these diseases (13). While we do not yet know the mechanisms and involvement of these disappearing species in our health, we may have a fleeting time window of opportunity to do so prior to their complete disappearance in natural ecosystems.

As with global warming on our planet, we have begun showing how chronic environmental changes in our gut can drive living organisms to vanish. We still know so little about basic effects of the physical environment on our microbiota, and yet they shape our health and wellbeing in countless ways. Mirroring traditional ecology, where pH, temperature, and other factors have always been at the very heart of understanding, my goal is to bring these fundamentals to the forefront of microbiome science as we seek to understand, predict, modify, and, importantly, safeguard the incredible microbial ecosystem we harbor within us. We were lucky to catch Muribaculaceae before it disappeared—what else are missing that may be on the brink of extinction?
